# Down-regulated expression of OPCML predicts an unfavorable prognosis and promotes disease progression in human gastric cancer

**DOI:** 10.1186/s12885-017-3203-y

**Published:** 2017-04-14

**Authors:** Xiangbin Xing, Weibin Cai, Sanmei Ma, Yongfei Wang, Huijuan Shi, Ming Li, Jinxia Jiao, Yang Yang, Longshan Liu, Xiangliang Zhang, Minhu Chen

**Affiliations:** 1 Department of Gastroenterology, the First Affiliated Hospital of Sun Yat-sen University, 58 Zhongshan II Road, Guangzhou, 510080 China; 2 Department of Biochemistry, Zhongshan Medical School, Sun Yat-sen University, Guangzhou, 510089 China; 3 Department of Biotechnology, Jinan University, Guangzhou, 510632 China; 4 Department of Pathology, the First Affiliated Hospital of Sun Yat-sen University, Guangzhou, 510080 China; 5 Department of Laboratory of General Surgery, the First Affiliated Hospital of Sun Yat-sen University, Guangzhou, 510080 China; 6 Department of Abdominal Surgery (Section 2), Affiliated Cancer Hospital of Guangzhou Medical University, Guangzhou, 510095 China

**Keywords:** OPCML, Gastric cancer, Prognosis, Progression

## Abstract

**Background:**

OPCML belongs to the IgLON family of Ig domain–containing GPI-anchored cell adhesion molecules and was recently found to be involved in carcinogenesis, while its role in gastric cancer remains unclear.

**Methods:**

We assessed expression and biological behavior of OPCML in gastric cancer.

**Results:**

OPCML expression was markedly reduced in tumor tissues and cancer cell lines. Decreased OPCML expression had a significant association with unfavorable tumor stage (*p* = 0.007) and grading (*p* < 0.001). Furthermore, the results revealed that OPCML was an independent prognostic factor for overall survival in gastric cancer (*p* = 0.002). In addition, ectopic expression of OPCML in cancer cells significantly inhibited cell viability (*p* < 0.01) and colony formation (*p* < 0.001), arrest cell cycle in G0/G1 phase and induced apoptosis, and suppressed tumor formation in nude mice. The alterations of phosphorylation status of AKT and its substrate GSK3β, up-regulation of pro-apoptotic regulators including caspase-3, caspase-9 and PARP, and up-regulation of cell cycle regulator p27, were implicated in the biological activity of OPCML in cancer cells.

**Conclusion:**

Down-regulated OPCML expression might serve as an independent predictor for unfavorable prognosis of patients, and the biological behavior supports its role as a tumor suppressor in gastric cancer.

**Electronic supplementary material:**

The online version of this article (doi:10.1186/s12885-017-3203-y) contains supplementary material, which is available to authorized users.

## Background

Despite the recent decreasing trend in incidence, gastric cancer is still one of the most common carcinomas and the second leading cause of cancer-related death in the world, leading to an estimated 738,000 deaths in 2008 [1]. The majority of gastric cancer patients are diagnosed at an advanced stage and the limited options are available for the treatment. Though many efforts have been made in the management of gastric cancer, most patients still have a poor prognosis, with a 5-year survival rate less than 25% [1, 2]. Studies have shown that the development of gastric cancer is associated with molecular alterations comprising inactivation of tumor suppressors, such as p16, APC and E-cadherin, some of which could be applied as prognostic factors [3–6]. However, additional biomarkers that could be used as prognostic marker and potential treatment target of gastric cancer are needed to help predict and improve the prognosis of patients.

Opioid binding protein/cell adhesion molecule-like gene (OPCML), also designated as OBCAM, was initially identified from rat brains and found to possess a specific opioid-binding activity [7]. It was later found to be a member of the IgLON family of GPI-anchored cell adhesion molecules, consisting of protein-protein interaction domains, such as three ‘I’ set Ig domains [8, 9]. Studies show that OPCML is normally expressed in brain and ovary, and also expressed in heart, placenta, testis, kidney, liver, pancreas, colon and stomach [9, 10]. Recently, OPCML was found to be a candidate tumor suppressor, which inhibited tumor growth in ovarian cancer and some other cancers including prostate cancer [9, 11]. A recent small-sample study by Wang et al. revealed that OPCML expression was markedly decreased in primary gastric cancer tissues, in comparison with normal stomach tissues [10]. However,the clinical implications and biological functions of OPCML in the progression of gastric cancer remain unknown.

In this study, we for the first time investigated the association between OPCML expression and clinicopathological features as well as prognosis of cancer patients. We also explored the biological functions of OPCML in tumor progression, and the molecular mechanisms underlying its behavior in gastric cancer. The results suggest that decreased OPCML expression might predict poor prognosis and promote disease progression in gastric cancer.

## Methods

### Subjects and immunohistochemical analysis

Tissue samples were obtained from 30 gastric cancer patients who underwent surgical resection at the First Affiliated Hospital of Sun Yat-sen University between 2010 and 2014. These samples contained both the tumor and tumor-free locations and used to assess the differential expression of OPCML using immunohistochemical analysis. In addition, we included 133 patients undergoing gastrectomy in the First Affiliated Hospital of Sun Yat-sen University between 1995 and 2004. Survival data were available from these 133 patients, with a median follow-up time of 24.4 months. None of the patients was treated with radiotherapy or chemotherapy preoperatively. Tumors were classified according to guidelines of the International Union against Cancer [12]. All patients provided informed consent for collection of the tissue samples and publication of the related data. This study was approved by the Ethics Committee of the Sun Yat-sen University.

Immunohistochemical analysis of OPCML protein was conducted as previously described [13]. The degree of OPCML immunostaining was evaluated according to both the proportion of positively stained cancer cells and intensity of staining. The proportion of cancer cells was scored according to the following criteria: 0 (no positive cancer cells), 1 (<10% positive cancer cells), 2 (10–50% positive cancer cells), and 3 (>50% positive cancer cells). Intensity of staining was graded as follows: 0 (no staining); 1 (weak staining = light yellow), 2 (moderate staining = yellow brown), and 3 (strong staining = brown). Staining index was calculated as the proportion score × the staining intensity score. We assessed the expression of OPCML in gastric cancer specimens according to the staining index, which scores as 0, 1, 2, 3, 4, 6, and 9. The staining index score of 4 was applied as the cut-off to differentiate between low and high expression of OPCML. OPCML antibody was purchased from Abcam (Cambridge, UK). The positive and negative controls for immunostaining are applied to assess the specificity of OPCML antibody [Additional file 1]. Assessment of the pathological slides was performed by two experienced pathologists who were blinded to clinicopathological characteristics and clinical outcome of the patients.

### Semi-quantitative RT-PCR and quantitative PCR analysis

Total RNA was extracted using NucleoSpin RNA Kit (Macherey-Nagel GmbH, Germany) according to the manufacturer’s instructions. Reverse transcription PCR (RT-PCR) and quantitative PCR was carried out as previously described [14]. PCR primers for the human OPCML gene were as follows: sense: 5′-CCTAGGTCCTCTGAGCAACG-3′, antisense: 5′-GGTCAAGGTAGCAGGAGCAG-3′. Primer sequences for S12 were as follows, sense: 5′- GCATTGCTGCTGGAGGTGTAAT-3′, antisense: 5′- CTGCAACCAACCACTTTACGG-3′.

### Western blot analysis

Total protein of cells was extracted by lysis buffer and concentration determination was conducted using the DC protein assay method of Bradford (Bio-Rad, Hercules, CA). Twenty mg of protein was loaded and separated in 12% sodium dodecyl sulfate polyacrylamide gel electrophoresis (SDS-PAGE) gel and transferred to polyvinylidine difluoride membranes (Millipore, Bedford, MA). The antibodies used for the analysis are as follows, OPCML (Abcam, Cambridge, UK), cyclin-dependent kinase inhibitor 1B (p27), cleaved caspase 3, cleaved caspase 9, poly ADP-ribose polymerase (PARP), phospho-Protein Kinase B (AKT) (Ser473) and phosphor-glycogen synthesis kinase 3β (GSK3β) (Cell Signaling Technology, Inc., Danvers, MA), and GAPDH (Good Here Biotechnology, Hangzhou, P.R. China).

### Gastric cancer cell lines and 5-aza-2′-deoxycytidine treatment

Gastric cancer cell lines (SGC7901, BGC823, AGS, MKN28, NCI-N87, SNU1 and MKN45) were obtained from American Type Culture Collection (Manassas, VA) in 2011. The cell lines were last tested and authenticated via cell line STR genotyping assay in 6 months before the experiment ended. The cell lines were cultured in RPMI-1640 media. Treatment of cell lines (SGC7901, BGC823 and AGS) with 5-aza-2′-deoxycytidine was performed as previously described [15].

### DNA construct and stable transfection

The cDNA of human OPCML was inserted into pcDNA3.1 (Invitrogen, Carlsbad, CA), as previously described [16]. SGC7901 and BGC823 cells were transfected with OPCML-pcDNA3.1 and the empty vector pcDNA3.1 using Lipofectamine 2000 according to the manufacturer’s instructions (Invitrogen, Carlsbad, CA), and stable clones were then generated and confirmed as previously described [16].

### Bisulfite treatment of DNA and methylation-specific PCR (MSP)

Genomic DNA was extracted using NucleoSpin DNA Kit (Macherey-Nagel GmbH, Germany) according to the manufacturer’s instructions, and was analyzed by the methylation-specific PCR (MSP) posterior to bisulfite conversion, as previously reported [17]. MSP primers used for OPCML promoter was as follows, M, sense: 5′-CGTTTAGTTTTTCGTGCGTTC-3′, antisense: 5′-CGAAAACGCGCAACCGACG-3′; U, sense: 5′-TTTGTTTAGTTTTTTGTGTGTTTG-3′, antisense: 5′-CAAAACAAAAACACACAACAACA-3′.

### Cellular assay

Cell viability was examined using Cell Counting Assay Kit-8 (CCK-8) (YiYuan Biotech, Guangzhou, China), according to the manufacturer’s instructions. Anchorage-dependent and -independent growth was respectively assessed by colony formation assay and colony formation in soft agar, as previously described [16]. By flow cytometry, cell cycle assay was performed using propidium iodide staining, and apoptosis assay was conducted using annexin V-FITC/PI staining (BD Biosciences, Erembodegem, Belgium), as previously reported [16, 17].

### Tumor xenograft formation and in vivo experiment

All the experiments on mice were approved by the Sun Yat-Sen University Committee for Animal Research and conducted in accordance with the highest international standards of humane care in animal experimentation. Four-week-old male athymic BALB/c nude mice were purchased from Vital River Laboratories (Beijing, China). All mice had free access to sterilized food and autoclaved water. In vivo experiments were initiated posterior to 1 week of acclimatization. 1 × 10^7^ suspended SGC-7901 cells (in 0.1 ml phosphate-buffered saline) transfected with OPCML or empty vector were injected subcutaneously into the dorsal left flank of each mouse. Tumor size was measured at each third day for 4 weeks. Tumor volume (mm^3^) and tumor weight were determined as previously described [17].

### Statistical analysis

Student’s *t* test, One-way ANOVA, Mann-Whitney U test and X^2^ test were used to determine the statistical difference. The survival curves were plotted by the Kaplan–Meier method, and comparison of survival times was conducted using the log-rank test. All tests were two-sided, and a *P* < 0.05 was considered to be statistically significant. Statistical analysis was carried out using SPSS 16.0 and Graphpad Prism V4.0.

## Results

### The association between OPCML expression and clinicopathological characteristics and prognosis

**Fig. 1:**
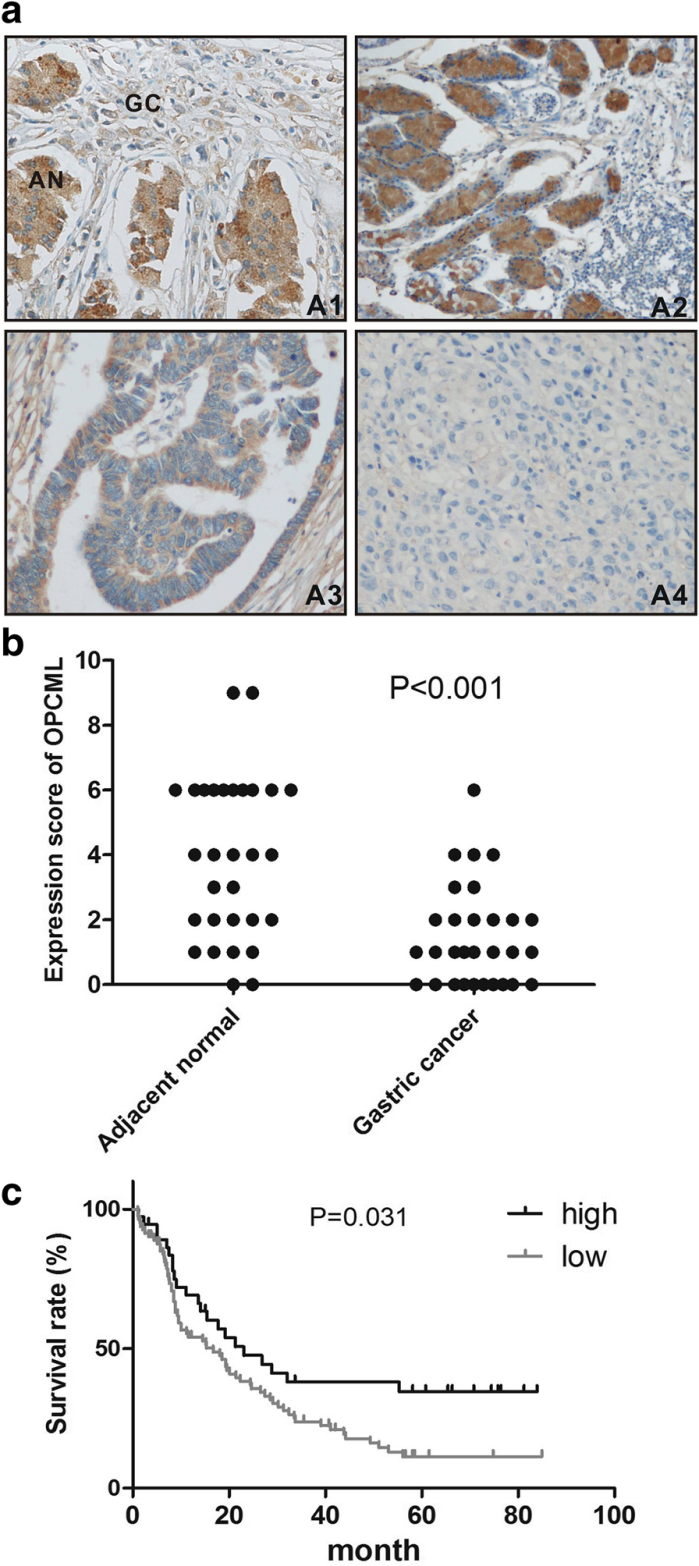
OPCML expression and its association with prognosis in gastric cancer. (**a**) Immunohistochemical images of OPCML protein expression in gastric cancer tissues and normal stomach tissues. The reduced expression of OPCML protein in cancerous tissues compared with adjacent non-cancerous tissues (A1, *upper-left panel*). OPCML protein expression was expressed in normal stomach tissues (A2, *upper-right panel*) and significantly down-regulated or lost in well-differentiated (A3, *lower-left panel*) and poorly-differentiated (A4, *lower-right panel*) tissues. (**b**) The significant reduction of OPCML protein expression in gastric cancer tissues as compared to the adjacent non-cancerous tissues by immnunohistochemistry (*P* < 0.001, *n* = 30). (**c**) Overall survival in patients with gastric cancer concerning OPCML expression. Kaplan–Meier curves displaying overall survival of patients stratified for OPCML expression. *P* value was calculated according to a log-rank test. AN:adjacent normal tissues; GC: gastric cancer tissues

In the first place, we performed immunohistochemistry to assess OPCML expression in 30 paired gastric cancer tissues and their adjacent normal gastric tissues. Abundant OPCML protein was easily detectable in non-cancerous gastric tissues and was shown to be majorly expressed in cell membrane and cytoplasm. Twenty-four of thirty patients exhibited the significantly decreased or lost OPCML expression in malignant tissues, as compared to the matched normal stomach tissues (Fig. 1 a1, b; *P* < 0.001).

Subsequently, we obtained tumor samples from 133 patients with adenocarcinoma of stomach and evaluated the differential expression of OPCML in gastric cancer, using the normal stomach tissues as control (Fig. 1 a2–4). Low expression of OPCML protein was exhibited in tumor tissues from 96/133 (72.2%) patients with gastric cancer (Table 1). Moreover, OPCML expression was found to be completely lost in samples from 45/133 (33.8%) gastric cancers. We subsequently analyzed the association between OPCML expression and clinicopathological characteristics of gastric cancer patients. Of note, tumors with more advanced tumor stages T3 and T4 tended to exhibit higher rates of low expression of OPCML compared with tumor stages T1 and T2 (Table 1, *P* = 0.007). Furthermore, OPCML expression was significantly associated with tumor grading, with poorly differentiated tumors possessing higher probability to demonstrate low expression of OPCML as compared to highly and moderately differentiated ones (Fig. 1 a3–4, Table 1, *P* < 0.001). Whereas, the results did not reveal the significant correlation between OPCML expression and other clinicopathological characteristics including age, gender, nodal status or state of metastasis (Table 1).

**Table 1:** Clinicopathological features and OPCML expression in 133 patients with gastric cancer

Variable	OPCML expression				*P* value
	Low	%	High	%	
All cases	96	72.2	37	27.8	
Age (years)					
>65	27	67.5	13	32.5	0.430
≤ 65	69	74.2	24	25.8	
Gender					
Male	74	69.2	33	30.8	0.115
Female	22	84.6	4	15.4	
Tumor stage					
T1/T2	12	50.0	12	50.0	0.007
T3/T4	84	77.1	25	22.9	
Nodal status					
N0/N1	32	69.6	14	30.4	0.625
N2/N3	64	73.6	23	26.4	
State of metastasis					
M0	60	73.2	22	26.8	0.747
M1	36	70.6	15	29.4	
Grading					
G1/G2	17	36.2	30	63.8	<0.001
G3	79	91.9	7	8.1	

We next assessed the correlation between OPCML expression and clinical outcome of patients with gastric cancer. As indicated in Fig. 1c and Table 2, OPCML expression exhibited a significant association with overall survival time of gastric cancer patients (*P* = 0.031). The patients with tumors of low OPCML expression had a significantly shorter mean overall survival time in comparison to patients with high OPCML expression (26.1 vs 39.7 months; Table 2). To assess whether OPCML expression is an independent prognostic factor for gastric cancer, we conducted a Cox multivariate regression analysis including age, tumor stage, nodal status, state of metastasis, grading and OPCML expression (Table 3). The results point to the independent prognostic significance of reduced OPCML expression in gastric cancer (HR = 2.34, 1.38–3.97; *P* = 0.002).

**Table 2:** The association of clinicopathological characteristics and survival of gastric cancer

	Cases	Events	Mean survival (months)	SE	Log-rank test (*p* value)
Age					
>65	40	32	27.0	3.9	0.726
≤ 65	93	70	30.5	3.2	
Tumor stage					
T1/T2	24	13	44.6	7.3	0.015
T3/T4	109	89	26.4	2.6	
Nodal status					
N0/N1	46	30	39.6	4.8	0.006
N2/N3	87	72	24.3	2.8	
State of metastasis					
M0	82	59	37.0	3.3	<0.0001
M1	51	43	17.0	3.0	
Grading					
G1/G2	47	32	36.1	4.9	0.082
G3	86	70	24.9	2.5	
OPCML expression					
Low	96	80	26.1	2.7	0.031
High	37	22	39.7	5.8

**Table 3:** Cox multivariate analysis of the prognostic factors for patients with gastric cancer

Variables	RR	95% CI	*p* value
Age (>65 vs ≤65)	1.23	0.77–1.79	0.34
Gender (male vs female)	1.47	0.80–2.65	0.21
Tumor stage (T3/T4 vs T1/T2)	2.34	1.38–3.97	0.002
Nodal status (N2/N3 vs N0/N1)	2.86	1.36–6.02	0.006
State of metastasis (M1 vs M0)	3.56	2.36–5.37	<0.00001
Grading (G3 vs G1/G2)	1.36	0.82–2.27	0.23
OPCML expression (low vs high)	2.34	1.38–3.97	0.002

### OPCML expression was significantly reduced or lost by promoter methylation in gastric cells

**Fig. 2:**
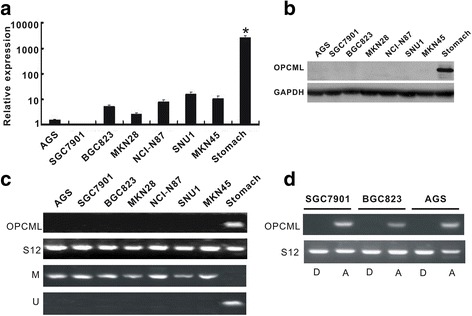
OPCML was decreased or lost by promoter methylation in gastric cells (**a**) and (**b**) The significant reduction of OPCML mRNA expression by real-time PCR and protein expression by western blot in seven gastric cancer cell lines, compared with normal stomach tissues. **c** OPCML mRNA expression by RT-PCR and promoter methylation by MSP in gastric cancer cell lines. **d** Changes of OPCML mRNA expression in gastric cancer cell lines after 5-aza-2′-deoxycytidine treatment. D: DMSO; A: 5-aza-2′-deoxycytidine. * *P* < 0.0001

We detected the expression of OPCML in seven gastric cancer cell lines. The results showed that expressions of both OPCML mRNA and protein were markedly down-regulated or lost in all seven gastric cancer cell lines, while it is readily detectable in the normal stomach tissues (Fig. 2a, b).

Using MSP, we determined the promoter methylation of OPCML in these seven gastric cancer cell lines. Interestingly, OPCML promoter was methylated in all seven cell lines, while not in the normal stomach tissues (Fig. 2c). Next, the expression changes of OPCML mRNA were analyzed in three cell lines (SGC7901, BGC823, AGS) after incubation with the methylation inhibitor 5-aza-2′-deoxycytidine. As shown in Fig. 2d, all these three cell lines exhibited restored mRNA levels of OPCML posterior to the treatment with 5-aza-2′-deoxycytidine. These data suggested the significant correlation of OPCML mRNA expression and promoter methylation.

### OPCML suppressed the growth of gastric cancer cells

**Fig. 3:**
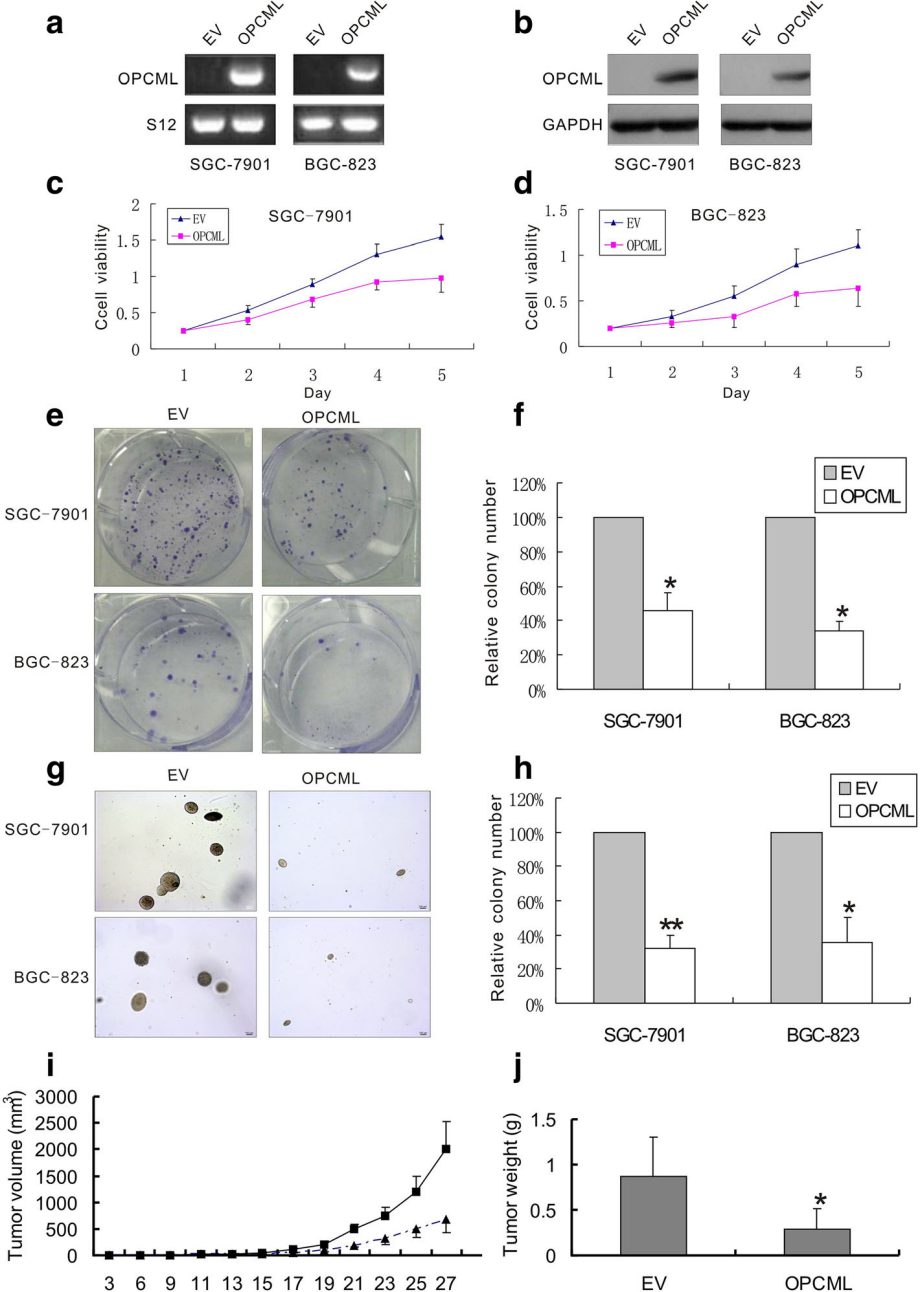
OPCML suppressed the growth of gastric cancer cells. **a** and **b** OPCML mRNA and protein expression in SGC-7901 and BGC-823 cells after transfected with OPCML plasmid. **c** and **d** effect of ectopic expression of OPCML on cell viability of SGC-7901(*P* < 0.01) and BGC-823 (*P* < 0.01) cells. **e** and **f** effect of ectopic OPCML expression on anchorage-dependent colony formation of SGC-7901 and BGC-823 cells. **g** and **h** effect of ectopic OPCML expression on anchorage-independent colony formation of SGC-7901 and BGC-823 cells. **i** and **j** effect of ectopic OPCML expression on the growth of SGC-7901 cells in vivo. Data are mean ± SE. of 5 independent experiments. * *P* < 0.001, ** *P* < 0.0001, versus empty vector. EV, empty vector; OPCML, OPCML-pcDNA 3.1

To understand the function of OPCML gene in gastric cancer, we assessed the effect of ectopic expression of OPCML on the growth of SGC-7901 and BGC-823 cells in which OPCML gene was silenced. As shown by RT-PCR and western blot, OPCML was significantly over-expressed in SGC-7901 and BGC-823 cells after stably transfected with OPCML-pcDNA3.1 plasmid (Fig. 3a, b). Representative clones of SGC-7901 and BGC-823 transfectants were cultivated and assayed for their viability using CCK-8 assay. Ectopic expression of OPCML resulted in an approximately 36% (*P* < 0.01) and 42% (*P* < 0.01) decrease of cell viability in SGC-7901 and BGC-823 cells at the 5th day of experiment, respectively (Fig. 3c, d). Colony formation assay revealed that the number of colonies formed by OPCML transfectants were significantly fewer compared with empty vector transfectants. OPCML SGC-7901 and BGC-823 transfectants exhibited an approximately 54% (*P* < 0.001) and 65% (*P* < 0.001) reduction in colony formation respectively, in comparison to empty vector–transfected control cells (Fig. 3e, f). Soft agar assay further confirmed the significant suppressive effect of OPCML on the growth of SGC-7901 (*P* < 0.0001) and BGC-823 cells (*P* < 0.001) (Fig. 3g, h).

We further examined whether OPCML inhibited the growth of gastric cancer cells in vivo. As indicated in Fig. 3i, OPCML-pcDNA 3.1 transfection led to the significant reduction of the tumor volume formed by SGC-7901 cells in nude mice (*P* < 0.01). When the in vivo experiment was concluded till the 27th day, mice with OPCML transfectants had a 66.7% decrease in tumor weight as compared to the empty vector (Fig. 3j, *P* <0.001).

### OPCML arrested cell cycle and induced cell apoptosis

**Fig. 4:**
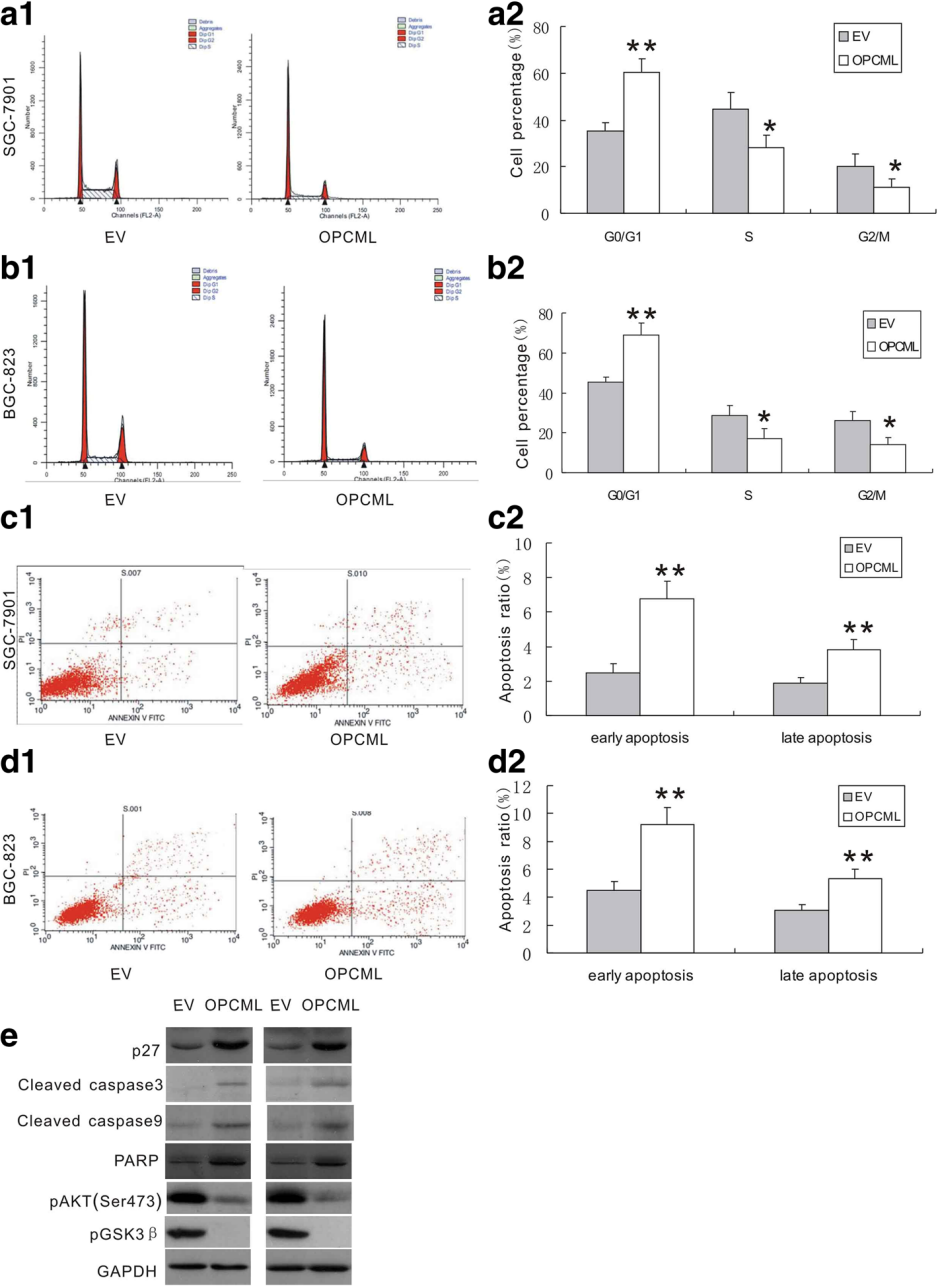
OPCML arrested cell cycle and induced apoptosis of gastric cancer cells. **a**1 and **b**1 Representative images of cell cycle distribution of SGC-7901 (**a**1) and BGC-823 (**b**1) cells. **a**2 and **b**2 Statistical analysis of the distribution percentage of cells in G0/G1, S, G2/M phases of SGC-7901 (**a**2) and BGC-823 (B2) cells. **c**1 and **d**1 Representative images of apoptosis of SGC-7901 (**c**1) and BGC-823 (**d**1) cells. **c**2 and **d**2 Statistical analysis of early apoptosis and late apoptosis ratio of SGC-7901 (**c**2) and BGC-823 (**d**2) cells. (Data are mean ± SE, versus empty vector; *n* = 5 independent experiments in triplicate). **e** changes of protein expression of G1/S phase transition regulator and the active form of pro-apoptosis regulators, as well as the phosphorylation levels of AKT and GSK3β in SGC-7901 and BGC-823 cells. ** *P* < 0.01,* *P* < 0.05

The following cell cycle analysis by flow cytometry indicated that, after trasnfected with OPCML-pcDNA3.1 plasmid, an elevated percentage of both SGC-7901(from 35.5% to 60.5%, *P* < 0.01) and BGC-823 (from 45.3% to 68.8%, *P* < 0.01) cells accumulated in the G0/G1 phase, as compared to cells transfected with empty vector (Fig. 4a, b). While ectopic expression of OPCML led to a decreased proportion of cell population of both SGC-7901 and BGC-823 cells at S and G2/M phase (all *P* < 0.05) (Fig. 4a, b). These results revealed that OPCML suppressed proliferation of gastric cancer cells by arresting cell cycle in the G0/G1 phase.

Because apoptosis was also frequently associated with cell growth inhibition by tumor suppressor, Annexin V-FITC/PI flow cytometric analysis was used to determine the effect of ectopic OPCML expression on apoptosis of SGC-7901 and BGC-823 cells. The analysis demonstrated a significant increase of cell population of both early apoptosis (*P* < 0.01) and late apoptosis (*P* < 0.01) in SGC-7901 cells transfected with OPCML-pcDNA 3.1, as compared to empty vector transfectants (Fig. 4c1, c2). Similar results were indicated in OPCML transfected BGC-823 cells, with a significant elevation of the percentage of both early apoptotic cell population (*P* < 0.01) and late apoptotic population (*P* < 0.01), compared with cells transfected with empty vector (Fig. 4d1, d2).

We further analyzed the expression of genes implicated in cell cycle arrest and apoptosis induction. Western blot was used to assess the expression of p27, an important regulator involved in transition checkpoint of G1 to S phase, and the expressions of the pro-apoptotic regulators, encompassing the active form of caspase-3, caspase-9 and nuclear enzyme poly (ADPribose) polymerase (PARP). As shown in Fig. 4e, expression of p27 protein was significantly up-regulated in both SGC-7901 and BGC-823 cells by ectopic OPCML expression. Moreover, expressions of activated form of caspase-3 and caspase-9, and PARP were markedly elevated in SGC-7901 and BGC-823 cells by OPCML (Fig. 4e). To investigate whether the activity of AKT was associated with the effects of OPCML on proliferation and apoptosis, we determined the phosphorylation status of AKT posterior to OPCML plasmid transfection. The results showed that AKT was constitutively activated in the two gastric cancer cell lines, and the phosphorylation level of AKT was significantly decreased by ectopic expression of OPCML. We next determined the effect of OPCML transfection on the phosphorylation status of the AKT downstream target, GSK3β. The constitutive phosphorylation of GSK3β was present in gastric cancer cells and its phosphorylation level was shown to be markedly reduced by ectopic OPCML expression (Fig. 4e).

## Discussion

In the current study, we showed that OPCML was expressed in the normal stomach while markedly down-regulated or lost in gastric cancer. We first used immunohistochemical assay to directly compare the expression of OPCML in gastric cancer tissues and their adjacent normal tissues in 30 gastric cancer patients. OPCML protein was shown to be significantly reduced in gastric cancer tissues, while readily expressed in adjacent normal stomach tissues. Next, we assessed the differential expression of OPCML in tumor samples from 133 patients with gastric cancer. The results revealed that the expression of OPCML was reduced in tumor samples from 96/133(72.2%) patients with gastric cancer and was completely lost in tumor tissues from 45/133 (33.8%) gastric cancers. A small-sample study by Wang et al. demonstrated a reduced expression of OPCML in primary gastric cancer tissues, compared with normal stomach tissues, similar to the finding in our study [10].

Moreover, we evaluated the association of OPCML expression and clinicopathological characteristics and prognosis in gastric cancer. Notably, the decrease of OPCML expression was significantly more frequent in the advanced stages of gastric cancer, in comparison to the early stages. In addition, poorly differentiated cancers exhibited a significantly lower expression of OPCML protein than well and moderately differentiated ones. Interestingly, gastric cancer patients with decreased OPCML expression possessed a significantly shorter survival as compared to cancers with preserved OPCML expression. Further multivariate regression analysis revealed that reduced OPCML expression is an independent predictor for poor outcome of gastric cancer patients. Taken together, these data revealed that reduction of OPCML expression might have the important clinical significance in the progression of gastric cancer.

In addition, our study showed that OPCML protein and mRNA were significantly decreased or lost in all 7 gastric cancer cell lines, as compared to the normal stomach tissues. Given the significant association between reduction of OPCML expression and gene methylation [9, 11], we analyzed the methylation status of OCPML promoter in these cell lines. The results showed that the silencing of OPCML gene was significantly correlated with promoter methylation. Furthermore, we also assessed the expression of OPCML mRNA after incubation with the methylation inhibitor 5-aza-2′-deoxycytidine in three cell lines. OPCML mRNA was found to be restored in all three cell lines, hence further confirming the link between the loss of OPCML mRNA expression and gene methylation.

Next, our study explored the effect of OPCML on the growth of gastric cancer cells in vitro and in vivo. The results revealed that ectopic expression of OPCML led to the inhibition of cell viability, and the suppression of both anchorage-dependent and -independent growth colony formation of gastric cells. Besides, the suppressive effect of OPCML on the growth of gastric cancer cells was confirmed in the subsequent experiments on nude mice. Sellar et al. reported that OPCML exhibited growth inhibitory activity in epithelial ovarian cancer, similar to the tumor-suppressive effect of OPCML indicated in our study [9]. Consistently, the in vitro study by Cui et al. also revealed that ectopic expression of OPCML suppressed tumor cell clonogenicity of prostate cancer and colon cancer cell lines [11]. Further analysis by flow cytometry showed that OPCML arrested gastric cells in the G0/G1 phase of the cell cycle. Also, OPCML was demonstrated to increase both early and late apoptosis in these cell lines. To our knowledge, no evidence has so far been found regarding the impact of OPCML on cell cycle and apoptosis of cancer cells. Taken together, these data thus suggest the role of OPCML as a candidate tumor suppressor in gastric cancer.

The possible molecular mechanism underlying OPCML arresting cell cycle in G0/G1 phase and inducing cell apoptosis was also investigated. In this study, p27 was found to be up-regulated in both SGC7901 and BGC823 cell lines by ectopic expression of OPCML. The transitions of cell cycle are strictly controlled by a series of cyclins, CDKs and inhibitory proteins. p27 possesses the inhibitory activity of CDK4 and CDK6, which phosphorylate Rb to promote the transition of G1 to S phase [18, 19]. In addition, ectopic OPCML expression led to the increase of activated form of caspase 9 and caspase 3. Caspase 9 is the crucial regulator of mitochondria-dependent apoptotic pathway, the activation of which launch the intrinsic apoptosis process via activating downstream apoptosis-associated molecules including caspase-3 and PARP.

This study revealed that AKT and its downstream target GSK3β were constitutively phosphorylated in the two gastric cancer cell lines and their phosphorylation levels were markedly suppressed by ectopic expression of OPCML. AKT has been shown to play an important role in the development and progression of numerous cancers through promoting cell proliferation and suppressing apoptosis [20, 21]. Constitutively phosphorylated AKT has been reported in some cancers, and activated AKT promotes cell proliferation and induces apoptosis via phosphorylating a number of transcription factors including GSK3β [22]. The findings in this study suggested that AKT/GSK3β signaling pathway might be implicated in the tumor-suppressing function of OPCML in gastric cancer.

## Conclusions

In conclusion, our study revealed that reduced expression of OPCML might have a significant correlation with unfavorable tumor stage and differentiation, and might independently predict poor prognosis of patients with gastric cancer. OPCML had a tumor-suppressing activity possibly via AKT/GSK3β signaling in gastric cancer [23].

## Additional file

Additional file 1: The specificity of OPCML antibody was determined in the immunohistological staining for OPCML protein expression. Normal stomach tissue (A) was used as positive control. System control (B) and isotype control (C) were also included as negative controls in normal stomach tissue. (TIFF 2899 kb)

## Abbreviations

AKT: Protein kinase B
GSK3β: Glycogen synthesis kinase 3β
OPCML: Opioid binding protein/cell adhesion molecule-like gene;
p27: Cyclin-dependent kinase inhibitor 1B.
PARP: Poly ADP-ribose polymerase
